# An Efficient Approach for Preprocessing Data from a Large-Scale Chemical Sensor Array

**DOI:** 10.3390/s140917786

**Published:** 2014-09-24

**Authors:** Marco Leo, Cosimo Distante, Mara Bernabei, Krishna Persaud

**Affiliations:** 1 National Research Council of Italy, Institute of Optics, via della Libertà 3 Arnesano (Lecce), 73010, Italy; E-Mail: cosimo.distante@cnr.it; 2 School of Chemical Engineering and Analytical Science, The University of Manchester, Oxford Road, Manchester M13 9PL, UK; E-Mails: mara.bernabei@manchester.ac.uk (M.B.); krishna.persaud@manchester.ac.uk (K.P.)

**Keywords:** large scale chemical sensor, data reduction, EigenOdour, pattern recognition

## Abstract

In this paper, an artificial olfactory system (Electronic Nose) that mimics the biological olfactory system is introduced. The device consists of a Large-Scale Chemical Sensor Array (16, 384 sensors, made of 24 different kinds of conducting polymer materials) that supplies data to software modules, which perform advanced data processing. In particular, the paper concentrates on the software components consisting, at first, of a crucial step that normalizes the heterogeneous sensor data and reduces their inherent noise. Cleaned data are then supplied as input to a data reduction procedure that extracts the most informative and discriminant directions in order to get an efficient representation in a lower dimensional space where it is possible to more easily find a robust mapping between the observed outputs and the characteristics of the odors in input to the device. Experimental qualitative proofs of the validity of the procedure are given by analyzing data acquired for two different pure analytes and their binary mixtures. Moreover, a classification task is performed in order to explore the possibility of automatically recognizing pure compounds and to predict binary mixture concentrations.

## 1. Introduction

The biological olfactory system is a very sophisticated system exhibiting unequaled sensitivity and discriminatory power [1] provided by millions of years of evolution.

Great progress has been achieved in the understanding of biological olfaction, inspiring new electronic nose developments. The olfactory system of animals can be divided into three basic blocks: Olfactory epithelium (OE), Olfactory bulb (OB) and Olfactory cortex (OC). The OE is the interface between the olfactory systems and the external environment. Different odours interact with different but overlapping sets of receptors present in the OE, generating different odor patterns. The odor response patterns produced in the OE are then processed by the OB and sent to the OC where they are further processed to achieve discrimination and quantification of odor stimuli. It is believed that the sensory characteristics observed in biological olfaction are due to the great number of receptor neurons, to their high level of redundancy and to the unique architecture of the olfactory pathway, where three main elements, the olfactory epithelium, the olfactory bulb and the olfactory cortex, are completely integrated [2].

Electronic nose technology has not been able to mimic these aspects of sensor redundancy and synergistic behavioral features. Current chemical sensor arrays, either homogeneous or heterogeneous, have few sensing elements when compared to natural noses. Many factors have limited the development of an artificial system having a great number of different sensing elements. So far, a large sensor array has implied unmanageable numbers of electrical connections, high power consumption, long response times, large instruments and, in consequence of all the previous factors, reduced portability of the system. The first electronic nose was built in 1982 by Persaud and Dodd [3]. It was composed of three gas sensors and its architecture is the same when implemented in actual systems. From that original device, the expression electronic nose was used in conferences and papers. Since the introduction of artificial olfaction, great progress has been made using this approach and such systems have been applied successfully in many areas, for example in industry [4], for controlling the production chain [5–7], and in environmental [8] and food [9] freshness control. Their potential in medical field as early and non-invasive diagnostic tool has also been demonstrated [10,11]. However, no device can ever guarantee performance, at least in part, comparable with biological systems as long as a number of overlapping receptors is used to generate the odors' patterns [12]. To this end, recently in [13] the fabrication of a device containing three sensor arrays of 300 elements using 24 different sensitive materials was reported. Recently, a large-scale chemical sensor array (65, 536 sensors) was introduced in [14].

The above recent pioneering works have introduced in the chemical sensor research community the problem of how to efficiently process the large amount of available sensory data. Traditional data analysis techniques used in this field are in fact inadequate to face this new challenge since they require methodological and computational constraints that can no longer be satisfied [15]. Hence, new and competent strategies need to be introduced to overcome the limits of the conventional approaches [16]. Many efforts have been made in other research fields (image processing, economics, medical) to build effective computational paradigms able to reduce the amount of data. In particular, Principal Component Analysis (PCA) [17] and Linear Discriminant Analysis (LDA) [18] are computationally efficient linear approaches used to reduce dimensionality reduction in various biological and industrial applications. Recently, the combination of PCA and LDA [19] has also demonstrated a great capability to embed information useful for the following classification task. As an alternative, non-linear strategies can be used. Kernel methods are an extension of linear strategies: canonical algorithms are reformulated in terms of Gram matrices, then generalized to nonlinear problems by substituting a kernel function to the inner product. Unfortunately the choice of the most appropriate kernel depends on the problem to be solved and, moreover, satisfying results can be achieved only after a fine tuning of its parameters. Manifold learning is an alternative approach to non-linear dimensionality reduction that has been widely exploited in pattern recognition, data analysis, and machine learning: algorithms for this task are based on the idea that the dimensionality of many data sets is only artificially high [20] and then a topological space that is locally Euclidean (manifold) can be searched to represent the initial data. Generative topological mapping (GTM) [21], locally linear embedding (LLE) [22], and ISOMAP [23] are some of the most used methods for manifold searching. In addition to the computational load needed, these methods generally only perform well when there is little or no noise. In the light of the above computational and implementation drawbacks, in the case of a large-scale sensor array, non-linear strategies result inappropriate since the acquired data are noisy, heterogeneous and sparse. On the other hand, linear techniques are not able to accomplish the data reduction task since there may be nonlinear relationships in the initial variables that would be ignored in the new data representation. Last but not least, a large-scale sensor array is, in general, a high-dimensional setting where the number of features of the given data is significantly greater than the number of observations (measurements) and then most of the aforementioned approaches cannot be exploited in their canonical implementation.

This paper tries to overcome the aforesaid limitations by introducing a data processing technique that combines modified versions of the canonical PCA and LDA in order to better embed information into the new data representation: the idea is to find the discriminant directions (by LDA) among those representing most of the variance of the initial data (as emerging by the Karhunen-Lo*è*ve transform). The alternative implementations of the standard techniques are used in order to estimate the best representation space even in the case of a few number of measurements. Graphical and numerical evidence of the effectiveness of the proposed solution is given on large sensory data acquired for two different pure analytes (Butanone and Ethanol) and their binary mixtures. Data were acquired by a realistic artifact of the olfactory epithelium that has been developed as a large sensor array mimicking some features of biological olfactory receptor neurons (ORNs). The sensor array is composed of four smaller arrays of 64 × 64 sensing elements deposited on a borosilicate substrate, giving a total of 16,384 sensors (with organic conductive polymers chosen as active sensing materials). The rest of the paper is organized as follows: Section 2 describes the large scale sensor array used whereas Section 3 details the processing techniques introduced to get an effective data reduction. The experimental setup is described in Section 4 whereas experimental proofs are reported in Section 5 and then discussed in Section 6. Finally, conclusions are given in Section 7.

## 2. Overview of the Electronic Device

In this section, the artificial olfactory system developed to generate data emulating the sensor inputs of the biological olfaction is briefly described. More details can be found in [14,24].

The hardware comprises the large sensor array and custom electronics designed and built to acquire the sensor response. A modular solution was adopted for the system. Hence, instead of building a unique large array of 65,536 elements, 16 sub-arrays of 4096 sensors were developed and the scanning system was partitioned into 16 acquisition modules capable of reading the smaller arrays simultaneously. Within each sub-array, the 4096 sensors are topologically and electrically arranged in a matrix of 64 rows and 64 columns. These are addressed using two multiplexers. The acquisition rate is independent of the number of sensors and the entire system is read at 1 s intervals. Conducting polymers were selected as sensing materials, as they show broad and overlapping specificity to many odors and their sensitivity and selectivity can be tuned making available a large number of sensing elements. Moreover, conducting polymers show reversibility, fast responses and sensitivity to many vapors, such as esters, alcohols and aromatic compounds [25]. Those characteristics make conducting polymers suitable for our aim of mimicking the ORNs in the olfactory bulbs. Twenty-five conducting polymers were deposited on the sensor arrays. They belong to the three main families of conducting polymers, that is polyaniline, polypyrrole and polythiophene and their sensitivities were differentiated by means of functional groups chains attached to the main ring, by using a range of dopant species or preparing copolymers (see Table 1).

The deposition of the sensing materials on the sensor die was carried out by airbrush techniques and using seven metallic masks to define seven different deposition region on each sensor array. Tuning experiments were performed to determine the optimal deposition parameters as the distance between the airbrush gun and the target array, the pressure of the atomizer gas and the amount of material sprayed per seconds. A sensor chamber for each sub-array was carefully designed for delivering the vapor analytes to the sensors while assuring homogeneous flux over all the sensors and avoiding recirculation regions within the chamber itself. The chamber is composed of a precisely machined piece of Teflon, acting as cover and having one inlet and one outlet, a silicone seal and a Teflon plate, which has the only aim of fixing the cover to the sensor board. A semi-automatic delivery system was developed and used for testing the array in a controlled environment. Details of the delivery system are given in the below Experimental Setup section.

A user - friendly interface software was developed using the Labview environment. It allows the user to control the functionality of the system, to visualize a real time colour-map of the sensor voltage and resistance (of one sensor board at time) and to display the trace of the percentage resistance, *R_s_*% (of ten selected sensors at a time). In particular the percentage resistance, *R_s_*% is given by

$$R_{s}\%=100*(R_{s}-R_{0})/R_{s}$$

where *R_s_* is the current resistance reading and *R*_0_ is the baseline resistance in clean air. The interface also consents the user to set the acquisition rate that, for all the measurements hereafter described, was set to 1 Hz.

## 3. An Approach to Handling Large Sensory Data Sets

Dealing with a large/scale array of chemical sensors introduces critical data handling issues. For this reason, the possibility of finding new representations of the data is desirable in order to concentrate the most of the information content in a reduced number of variables. Traditional data reduction techniques (such as Principal Component Analysis, Factor Analysis, *etc.*) generally require estimating the solutions of the linear/nonlinear problem. If the dimensionality of the variables increases, this can lead to a huge computational load. Moreover, classical approaches work well if the data are not sparse, while they are less efficient in the case of sparse data, as unfortunately the ones of the above described large scale array of chemical sensors tend to be. In fact the data acquired with the device described in the previous section and consisting of N = 16,384 conductive polymer sensors are generally sparse since not all the sensors respond to a substance and/or their responses kinetics are not synchronous.

Hereafter, the solution we adopted to process this large number of sparse data is shown. Let *T* be the number of samples for each sensors and *M* the number of measurement for each source *y*_1_, *y*_2_, …, *y_M_*. This leads to a three-dimensional data structure of dimension *M* × *N* × *T*.

**Figure 1.**
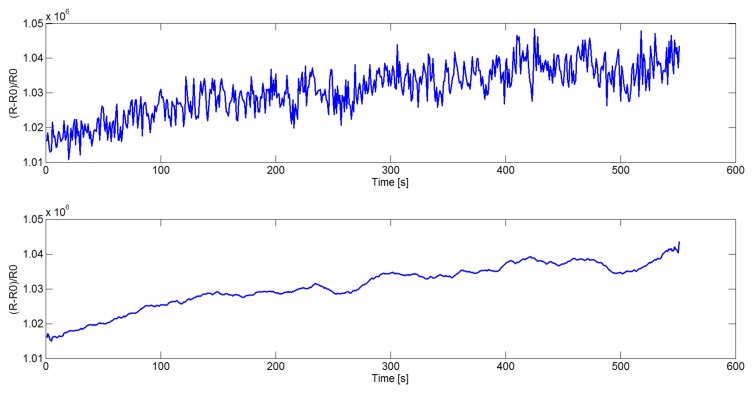
The original (**top**) and smoothed (**bottom**) signal acquired from one sensor in the device.

First of all, the sensor signals need to be filtered in order to reduce the noise. In this paper a Savitzky-Golay filter [26], instead of the mostly used moving average window [27], is introduced. Savitzky and Golay proposed a method of data smoothing based on local least-squares polynomial approximation. They demonstrated that least-squares smoothing reduces noise while maintaining the shape and the height of waveform peaks. In Figure 1 the original (top) and smoothed (bottom) signal acquired from one sensor in the device is reported.

This feature is fundamental to allow the following data reduction steps to perform well. In fact, after smoothing, the sensor response to a substance is generally calculated as the fractional variation of its resistance:

(1) $$\frac{\Delta R}{R}=\frac{R-R_{0}}{R_{0}}$$

In this equation, *R*_0_ is the sensor resistance at the beginning of the measurement (baseline) and *R* is the resistance at steady state. The use of as sensor response allows reduction of the effects of multiplicative drift. Finally, autoscaling of the sensor responses was performed in order to make all the sensors equally weighted. This sets the mean and the variance of all the sensor response distributions to zero and one respectively. At the end of this processing phase, oncoming data consist of a two-dimensional structure of dimensions *M* × *N*. At this point one of the standard data reduction techniques, for example Principal Component Analysis, could be applied. Defining the average measurement $\Psi =\frac{1}{M}\sum_{i=1}^{M}y_{i}$, each measurement differs from the average by vector Φ*_i_* = *y_i_* — Ψ. By applying PCA, the best representation of the data is obtained by seeking a set of *M* orthonormal vectors *u_i_* chosen such that

(2) $$\lambda_{k}=\frac{1}{M}\sum_{i=1}^{M}{\left(u_{k}^{T}\Phi_{i}\right)}^{2}$$

is a maximum subject to

(3) $$u_{l}^{T}u_{k}=\delta_{lk}=\{\begin{array}{cc} 1 & if\;l=k\\ 0 & \text{otherwise} \end{array}$$

The vectors *u_k_* and scalars *λ_k_* are the eigenvectors and eigenvalues, respectively, of the covariance matrix

(4) $$C=\frac{1}{M}\sum_{i=1}^{M}\Phi_{i}\Phi_{i}^{T}=AA^{T}$$

where the matrix *A* = [Φ_1_, Φ_2_,…, Φ*_M_*], represent the normalized measurement matrix.

The matrix *C* is of dimensionality *N* × *N*. The larger is N, the higher the computational load is required to compute its eigenvalues and eigenvectors. In the case of the considered sensory device the problem becomes an intractable task since *N* = 16, 384. Anyhow, since *M* ≪ *N*, there will be only *M* — 1, rather than *N*, meaningful eigenvectors and it is possible to compute them by solving the eigenvectors of a *M* × *M* matrix. This method comes from the well known Eigenface method introduced in [28] for face image recognition and here it is renamed *EigenOdours* since applied for the first time to the chemical sensor community. Let be *v_i_* the eigenvectors of *A^T^A* such that

(5) $$A^{T}A{\text{v}}_{i}=\mu_{i}{\text{v}}_{i}$$

then premultiplying both sides by *A* we have

(6) $$AA^{T}A{\text{v}}_{i}=\mu_{i}A{\text{v}}_{i}$$

where we notice that *A*v*_i_* are the eigenvectors of the original covariance matrix *C* = *AA^T^*. Following this analysis, we construct the *M* × *M* matrix *L* = *A^T^A* where the element of the matrix $L_{mn}=\Phi_{m}^{T}\Phi_{n}$ and find the M eigenvectors v_1_, of *L*. These vectors determine linear combinations of the M training set measurements to form the original EigenOdour *u_l_* given by

(7) $$u_{1}=\sum_{k=1}^{M}v_{1k}\Phi_{k}$$

At this point, we have v_1_,…, v_m_ eigenvectors (*i.e.*, EigenOdours) of dimension *K* × 1 where we may select first two or three of them in order to project data and inspect with a plot. Alternatively, we may also take all of them for recognition purposes. This is feasible since the computed EigenOdours represent the basis of a new vectorial space where it is possible to project the sensory measurements through a linear combination of them. Each EigenOdour accounts some part of the variance of the sensory data and it is orthogonal to (*i.e.*, uncorrelated with) the other components. The accounted variance decreases from the first extracted EigenOdour to the last one. The main advantage of this approach is the possibility of compressing the information content in a minimum number of variables and this is very advantageous for data storage and representation, allowing qualitative exploration of the data content. However, the above transformation is class-independent and so it is not fully suited to reach the main goal of the large scale array which is to mimic the biological olfactory epithelium, *i.e.*, to automatically classify the odors supplied to the sensors. To fully accomplish the data classification task it is then necessary to couple the EigenOdour analysis with a class-dependent transformation. To this end a modified version of the *Linear Discriminant Analysis (LDA)* [29] is proposed: it takes as input the data projected into the vectorial space defined by the EigenOdours and then it finds a linear combination of data that guarantees separability among classes by maximizing the ratio between-class variance to within-class variance in any particular data set. The intuition behind the method is to determine a subspace in which the data points of the original problem are “separable”. Separability is defined in terms of statistical measures of mean value and variance. The solution can be obtained by solving a generalized eigenvalue system (for this reason it cannot be performed directly on the initial large set of data) and this is done as explained below. Let *x*_1_,…, *x_m_* ∈ ℜ*^p^* be a set of *M* data samples belonging to *c* different class sets, where *p* is the number of retained directions after EigenOdours computation.

Let the between-class scatter matrix be defined as

$$S_{B}=\sum_{i=1}^{c}M_{i}(\mu_{i}-\mu ){\left(\mu_{i}-\mu \right)}^{T}$$

and the within-class scatter matrix be defined as

$$S_{w}=\sum_{i=1}^{c}\sum_{x_{k}\in X_{i}}(x_{k}-\mu_{i}){\left(x_{k}-\mu_{i}\right)}^{T}$$

where *μ_i_* is the mean image of class *X_i_* and *M_i_* is the number of samples in class *X_i_*.

In the standard *LDA*, if *S_W_* is nonsingular, the optimal projection *W_opt_* is chosen as:

(8) $$W_{\text{opt}}=\underset{x}{arg max}\frac{\left|W^{T}S_{B}W\right|}{W^{T}S_{W}W}=\left[w_{1}w_{2}\ldots w_{m}\right]$$

where {*w_i_*|*i* =1, 2, …*m*} is the set of generalized eigen-vectors of *S_B_* and *S_W_* corresponding to the *m* largest generalized eigenvalues {λ*_i_*|*i* =, 2,…, *m*}, *i.e.*,

$$\begin{array}{cc} S_{B}w_{i}=\lambda_{i}S_{W}w_{i}, & i=1,2,\ldots ,m \end{array}$$

In the case of the large scale array of sensors, where the number of measurements is lower than the dimensionality of data, the matric *S_W_* is always singular since the rank of *S_W_* is at most is *M* − *c*. In order to overcome the complication of a singular *S_W_*, an alternative to the criterion in Equation (9) is here proposed. The idea comes from the work in [30] and it is expressed as:

(9) $$W_{\text{opt}}^{T}=W_{\text{LDA}}^{T}W_{\text{EigenOdours}}^{T}$$

where

(10) $$W_{\text{EigenOdours}}=\underset{W}{\text{arg}\max}\left|W^{T}S_{T}W\right|$$

(11) $$W_{\text{LDA}}=\underset{x}{\arg\max}\frac{\left|W^{T}W_{\text{EigenOdours}}^{T}S_{B}W_{\text{EigenOdours}}W\right|}{\left|W^{T}W_{\text{EigenOdours}}^{T}S_{W}W_{\text{EigenOdours}}W\right|}$$

where

$$S_{T}=\sum_{k=1}^{M}\left(x_{k}-\mu \right){\left(x_{k}-\mu \right)}^{T}$$

After getting the data representation into the vectorial space defined by the aforesaid procedures, odors' data classification can be more easily performed by using a robust classifier. To accomplish this task, in this paper, the Multi-class Support Vector Machine (SVM) has been chosen. Support vector machines (SVMs) were originally designed for binary classification. How to effectively extend them for multi-class classification is still an ongoing research issue. Currently, there are two types of approaches for multiclass SVM [31]. The first one is based on the combination of several binary classifiers while the other works by searching a unique optimization formulation for all data. A method which considers all variables together has been proposed by Crammer and Singer [32] and it has been efficiently exploited in this paper.

Given *M* training data

$$\left(x_{1}^{\text{LDA}},y_{1}),\ldots ,(x_{M}^{\text{LDA}},y_{M}\right)$$

where: $x_{i}^{\text{LDA}}\in {ℜ}^{q}$ are the input q variables retained after LDA computation; *y_i_* ∈ {1, …*c*} are the labels of the *c* output classes, *i.e.*, the number of odors to be classified.

Basically the goal is to solve the following primary problem:

(12) $$\begin{array}{r} \underset{w_{m},\xi_{i}}{\min}\frac{1}{2}\overset{c}{\underset{m=1}{\text{∑}}}w_{m}^{T}w_{m}+C\overset{M}{\underset{i=1}{\text{∑}}}\xi_{i}w_{y_{i}}^{T}(x_{i}^{\text{LDA}})-w_{m}^{T}\phi (x_{i}^{\text{LDA}})\geq \ldots \\ \ldots \geq e_{i}^{m}-\xi_{i},i=1,\ldots ,l \end{array}$$

where:

$$\begin{array}{c} e_{i}^{m}\equiv 1-\delta_{y_{i},m}\\ \delta_{y_{i},m}\equiv \{\begin{array}{cc} 1 & \text{if}\;y_{i}=m\\ 0 & \text{if}\;y_{i}\neq m. \end{array} \end{array}$$

and *C* is the penalty parameter.

The solution is found through the following decision function:

$$\arg\underset{m=1,\ldots ,k}{\max}w_{m}^{T}\phi (x^{\text{LDA}})$$

The dual problem of 12 is:

(13) $$\begin{array}{lll} \underset{\alpha }{\min} & f(\alpha )=\frac{1}{2}\overset{M}{\underset{i=1}{\text{∑}}}\overset{M}{\underset{j=1}{\text{∑}}}K_{i,j}{\bar{\alpha }}_{i}^{T}{\bar{\alpha }}_{j}+\overset{M}{\underset{i=1}{\text{∑}}}{\bar{\alpha }}_{i}^{T}{\bar{e}}_{j}\overset{c}{\underset{m=1}{\text{∑}}}\alpha_{i}^{m}=0,\\ & i=1,\ldots \alpha_{i}^{m}\leq 0,\;\text{if}\;y_{i}\neq m\alpha_{i}^{m}\leq C,\;\text{if}\;y_{i}=m_{i}=1,\ldots .M, & m=1,\ldots ,c \end{array}$$

where $k_{i,j}=\phi (x_{i}^{\text{LDA}})$, ${\bar{\alpha }}_{i}\equiv {\left[\alpha_{i}^{1},\ldots ,\alpha_{i}^{k}\right]}^{T}$ and ${\bar{e}}_{i}\equiv {\left[e_{i}^{1},\ldots ,e_{i}^{k}\right]}^{T}$. The decision function is:

$$\arg\underset{m=1,\ldots ,c}{\max}\sum_{i=1}^{M}\alpha_{i}^{M}K(x_{i}^{\text{LDA}},x).$$

## 4. Experimental Setup

A chemical analyte delivery system based on bubblers coupled to mass flow controllers (MFC) for vapour dilution was used for rigorous testing of the sensor array This allowed the delivery of pure analytes and their binary mixtures over a broad range of different concentrations. Humidity and temperature sensors enabled the monitoring of these physical parameters that may affect the sensor response. During the experiments, in fact, both humidity and temperature were kept constant.

**Figure 2.**
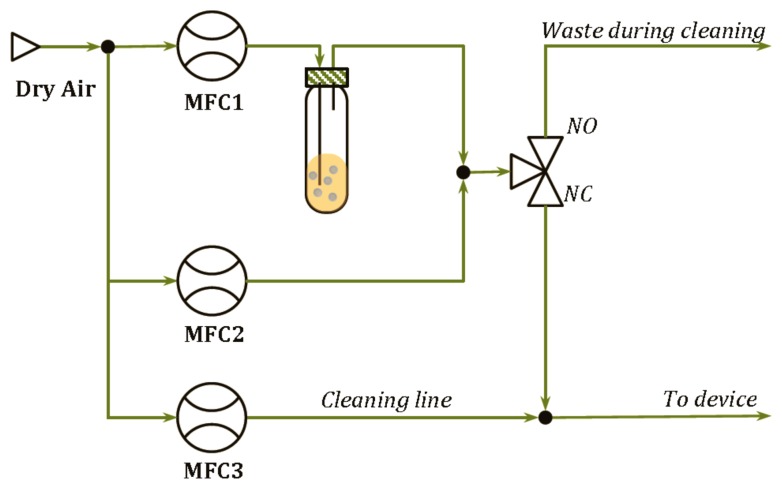
System set up for delivering low concentrations of one analyte.

**Figure 3.**
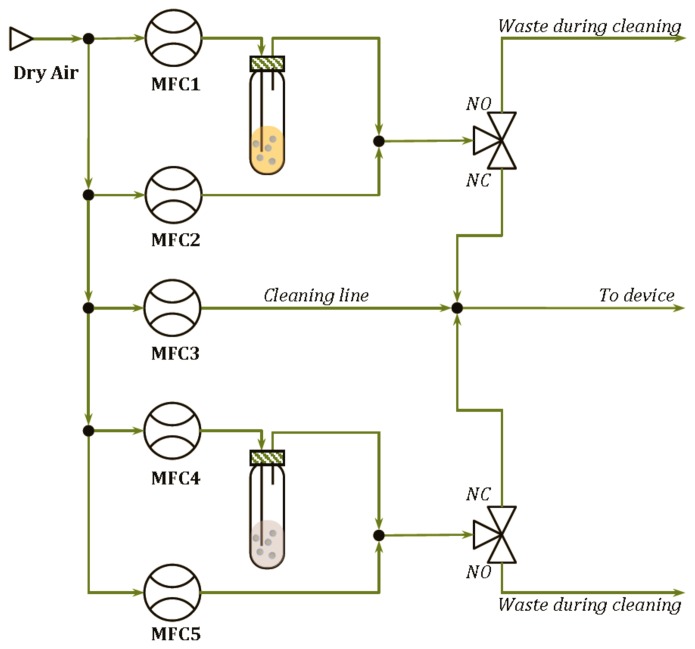
System set up for delivering binary mixtures.

The delivery modules developed are shown in Figure 2 for the pure analytes and in Figure 3 for the binary mixtures. In such systems, the Drechsel bottle contains the liquid sample and is kept at constant temperature. In this condition, a controlled flow of a carrier gas passing through the bottle causes the bubbling of the liquid, the formation of a steady headspace on the top of the liquid and the delivery of the saturated vapor out of the bottle. A second delivery line is then used to mix a fixed concentration of the carrier gas with the vapors coming from the Drechsel bottle, allowing the dilution of the saturated vapors. Hence, the desired concentrations of the analyte are generated and, in the case of the module shown in Figure 2, they are delivered to the device connected at the end of the delivery system. In the case of the module shown in Figure 3, the desired concentrations of two analytes are mixed together before their delivering to the device. In the two diagrams, a “cleaning” line is shown. The aim of this line is to deliver clean and dry air to the sensors during the cleaning phase that follows the measurement phase. It also permits the generation of the baseline, before the start of each measurement.

Using the system described in Figure 2, laboratory experiments were conducted and two datasets were generated by using the large scale array. Dataset 1 captured the sensor responses to two analytes (Ethanol and Butanone) and their binary mixtures for three different ratios. Starting from pure analyte, A for instance, the percentage of A in the mixture decreases, while the percentage of the odor B in the mixture increases until pure analyte B is reached. With this data set, a collection of odors with increasing similarity to an odor (e.g., A) and increasing dissimilarity to another odor (e.g., **B**) was obtained. Dataset 2 was acquired by exposing the gas sensors at different concentrations of an odor (butanone) with the constant presence of a background of another compound (ethanol). Tables 2 and 3 report some details of the experimental setup. In particular the left column indicates the number of measurement for each odor, the central column proportions of the two analytes (and relative concentration in parts per million, ppm) and the right column reports the labels that, hereafter, will be used to refer to each odor.

## 5. Experimental Results

**Figure 4.**
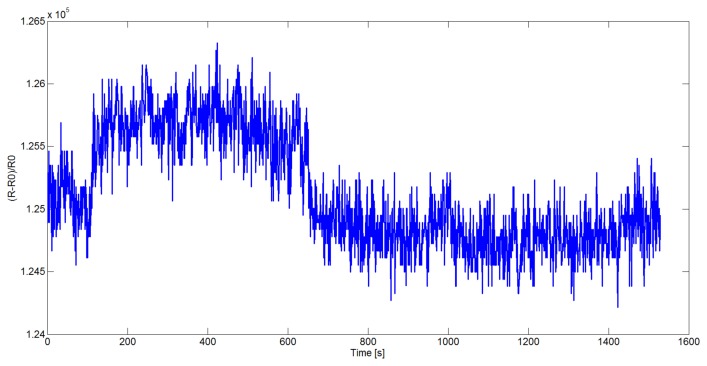
The output signal acquired in correspondence of the submission of Butanone (19,520 ppm).

In this section, the computational analysis of the data acquired by the large scale array is reported. In particular, the goal of the experimental phase was to verify the capability of the data reduction procedure described in Section 3 to preserve the informative content of the initial data and to supply a data representation in a new vectorial space where it's possible to easier find a robust mapping between the observed outputs and the characteristics of the odors in input to the device. The first experiment was performed on the Dataset 1 in Table 2. For each of the 41 measurements the considered analyte (or mixture) was presented to the device and the corresponding responses of the 16, 536 sensors were captured with a sampling frequency of 1*Hz*. All the substances were delivered after 100 s of baseline measurement in clean air, the recovery process was started after 650 s by again passing clean air through the system. In Figure 4 the data read from one sensor (on the board 1) when Butanone (19,520 ppm) was supplied as input is shown: the timing of insertion (*t* = 100) and cleaning (*t* = 650) of the device can be observed in the represented sensor resistivity curve.

After the acquisition phase, according to the procedure described in Section 3, data coming form the large array were smoothed by using the Savitzky-Golay filter (using a polynomial order *k* = 2 and a frame size *f* = 101) and then the fractional variation of the output resistance was computed for each sensor. At the end of these steps, the resulting 41 × 16, 536 data matrix was given as the input to the algorithm for the identification of the EigenOdours.

**Figure 5.**
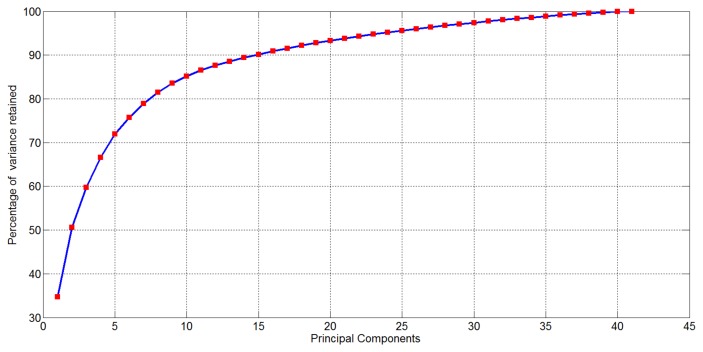
Percentage of cumulative variance of the initial data retained in the components of the new representation for the dataset 1.

After computing EigenOdours, the percentage of cumulative variance retained in the new representation is reported in Figure 5: notice that the 90% of the variance of the initial data representation (16, 536 variables) is accounted in the first 15 EigenOdours.

**Figure 6.**
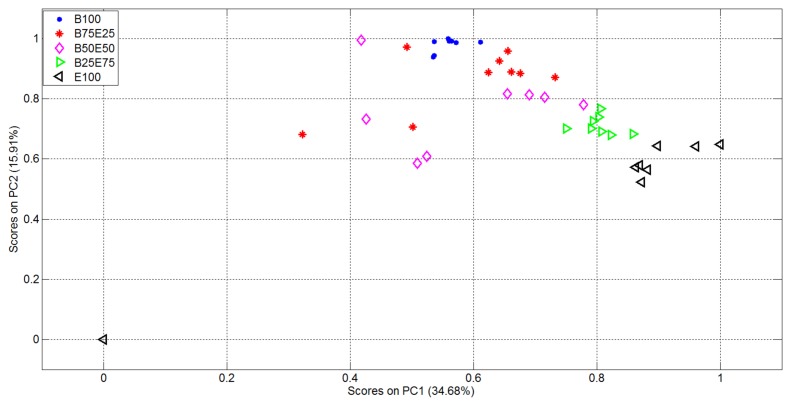
Representation of the Dataset1 measurements in the vectorial space identified by the two most relevant EigenOdours.

The plots of the first two in the vectorial space represented by the EigenOdours are then reported in Figure 6.

**Figure 7.**
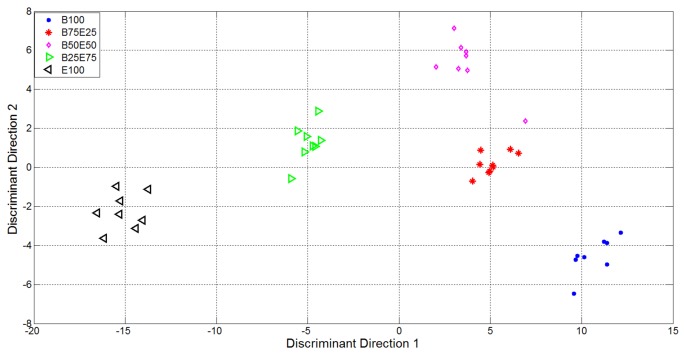
Representation of the data in the vectorial space identified by the first two discriminant directions obtained through the LDA approach.

From Figure 6 it is evident the formation of clusters related with the supplied analytes and mixtures. In particular the pure analytes (Butanone and Ethanol, represented using blue points and black triangles respectively) formed two highly distinct clusters, *i.e.*, with high internal compactness and external separability. Mixture representations were instead less separable. In particular, the internal variances of the mixtures with 50% of Ethanol and 50% of Butanone (purple rhombus) and 75% of Butanone and 25% of Ethanol (red star) are very high: it follows that the separability among clusters of gas mixtures was not optimal, *i.e.*, suitable for automatic classification of the substances given as input to the electronic device. In light of this, the variables relative to the representation of the first 15 EigenOdours were used as input to the Linear Discriminant Analysis, in order to get a class-dependent data representation. The plots of the first two variables represented in the discriminant components obtained are reported in Figure 7. It is quite evident how this new representation produces compact and well separated clusters.

It is quite evident how, in this new representation (built taking under consideration the label of classes for available measures), the clusters relative to mixtures of gases were also compact and all the clusters were separated.

In order to prove the effectiveness of the new representation in a context of automatic classification of substances a finally validation step was performed by considering a *K* – *fold cross* – *validation* approach (*k* = 10) and the multiclasses SVM algorithm described in Section 3. The goal of this step is to automatically recognize pure compound (B100 , *i.e.*, 100% Butanone and E100, i.e., 100% Ethanol) but also to predict the concentration of their binary mixtures (listed in Table 2). Table 4 reports the confusion matrix for the classification task: each row indicates the estimated labels and each column indicates the known labels.

Pure compounds were always correctly classified. Concerning the binary mixture prediction, in most of experimental proofs the SVM machine was able to correctly associate the data in input with the actual concentrations in input. The prediction failed in five cases: specifically, three mixtures obtained by combining 25% Butanone and 75% Ethanol where predicted as 50% Butanone and 50% Ethanol and two mixtures obtained by combining 50% Butanone and 50% Ethanol where predicted as 75% Butanone and 25% Ethanol. This leads to the following average Precision *P* and Recall *R* rates:

(14) $$\begin{array}{r} P=\frac{\text{TruePositive}}{\text{TruePositive}+\text{FalsePositive}}=\frac{P_{B100}+P_{B25E75}+P_{B50E50}+P_{B25E75}+P_{E100}}{5}=\\ \frac{\frac{8}{8}+\frac{8}{10}+\frac{7}{10}+\frac{5}{5}+\frac{8}{8}}{5}=\frac{1+0.8+0.7+1+1}{5}=0.9\\ P=\frac{\text{TruePositive}}{\text{TruePositive}+\text{FalsePositive}}=\frac{P_{B100}+P_{B25E75}+P_{B50E50}+P_{B25E70}+P_{E10}}{5}=\\ \frac{\frac{8}{8}+\frac{8}{8}+\frac{7}{9}+\frac{5}{8}+\frac{8}{8}}{5}=\frac{1+1+0.77+0.625+1}{5}=0.879 \end{array}$$

where

$$P_{x}=\frac{T_{p}(x)}{Tp(x)+Fp(x)}$$

and

$$R_{x}=\frac{Tp(x)}{Tp(x)+Fn(x)}$$

with *Tp*(*x*) representing true positive (odors correctly classified as belonging to the *x* class), *F_p_*(*x*) for false positive (odors erroneously assigned to the *x* class) and *F_n_* for false negative (odors of class *x* assigned to any other class).

**Figure 8.**
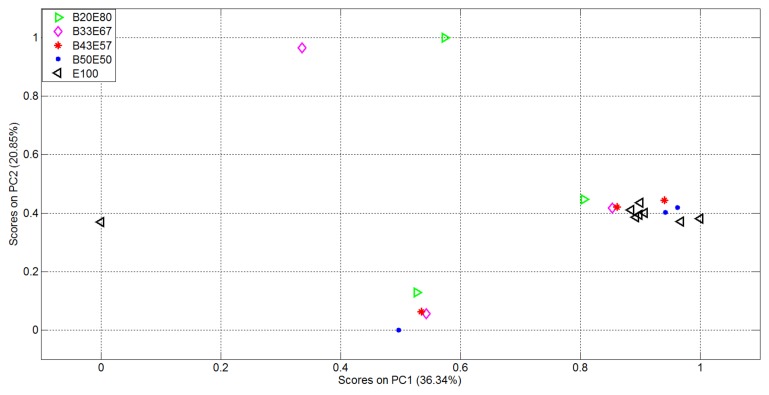
Representation of the Dataset 2 measurements in the vectorial space identified by the two most relevant EigenOdours.

The second experiment was then performed on the Dataset 2 detailed in Table 3. For each of the 20 measurements the considered mixture (and pure Ethanol) was inserted into the device and the corresponding responses of the 16, 536 sensors were analyzed as in experiment 1. The plots of the first two variables in the vectorial space represented by the EigenOdours are reported in Figure 8 where it's evident that clusters are not well distinguishable, neither for pure analyte nor for binary mixtures.

**Figure 9.**
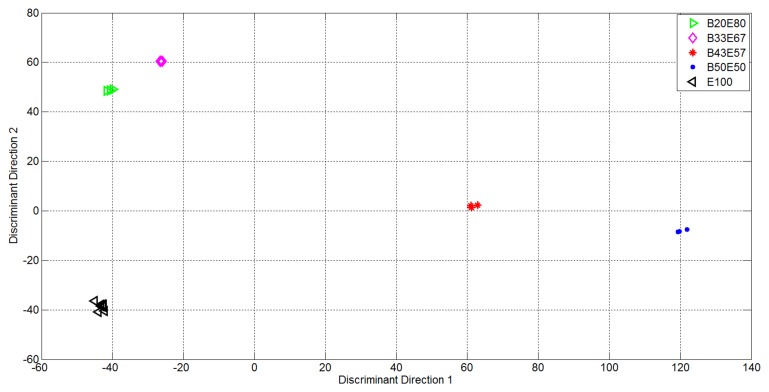
First 2 directions in the LDA representation.

The first 15 components of new representation were then used as input to the LDA approach. The plots of the first two components in the achieved LDA space are then reported in Figure 9.

It is evident that in this new representation, that considers the labels of the classes for available measurements, the data distribution is perfectly suited for prediction of of concentrations of binary mixtures. This was again proven by considering a *K* – *fold cross* – *validation* approach and the multiclasses SVM algorithm: Table 5 reports the confusion matrix representing the ability of proposed solution to accomplish the classification task. In the table each row indicates the estimated labels whereas each column indicates the known labels.

Binary mixture concentrations were automatically labeled: this is a very encouraging results considering that in this experiment the concentrations of the Ethanol remained constant making the prediction of the binary mixtures more difficult than in experiment 1. From experiments 1 and 2 it is possible to infer that using the proposed data reduction procedure the data acquired by a large scale array of sensor can be exploited for automatic substance classification. In particular the classification task can be performed at best if only the concentration of one of the two considered analytes varies (as in the experiment 2) but, even in case of variation of the concentrations of both analytes (as in the experiment 1) the classification accuracy (in terms of precision and recall) is very high.

## 6. Discussion and Comparison with Other Methods

This section discusses comparisons of the results achieved in the experimental phase with those of other researches describing mathematical and statistical methods for analyzing chemical sensor-array data of the same type, especially for conducting polymer e-noses. This goal has been reached by extracting the classification performance, on the same tested datasets and using the same classifier, that can be achieved by replacing the proposed strategy with some classical pre-processing approaches. In particular, four alternative ways of pre-processing the large scale data were compared with the proposed one:

- Raw Data
- Canonical Principal Component Analysis (PCA)
- Partial Least Square Regression Analysis (PLSRA)
- Fast Fourier Transform (FFT) [33]
- Discrete Wavelet Transform (DWT) [34]

**Figure 10.**
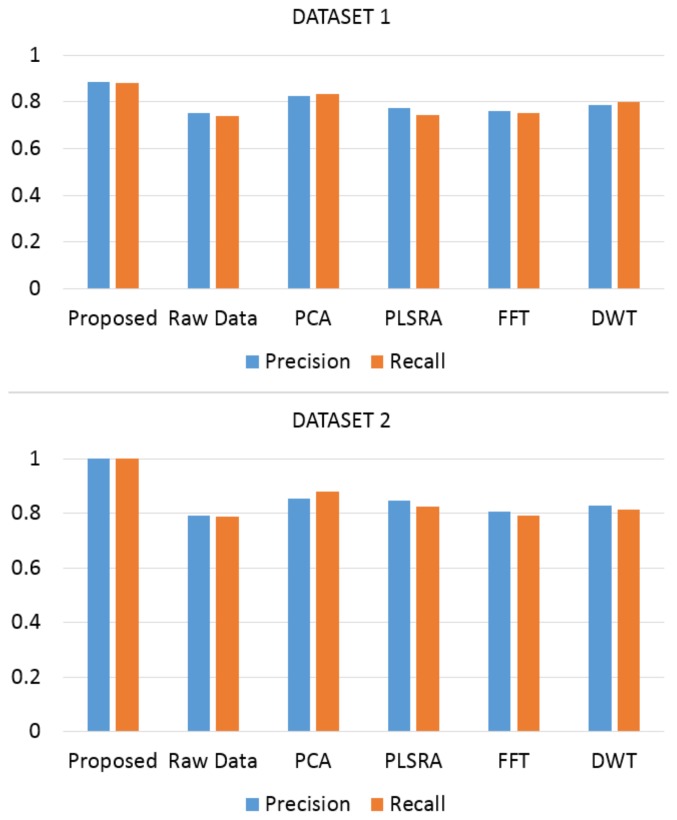
Performance comparison in terms of Precision and Recall. Multiclass SVM was used to classify input data.

Using Raw Data the two-dimensional structure coming from the initial smoothing of the signals and the subsequent application of the Equation (1) was given as input to the multiclass SVM classifier. Canonical PCA was computed using Eigenvalue Decomposition of the covariance matrix. PLSRA was instead implemented supplying as output block the butanone concentrations *Y* = [*C*_1_,…,*C_n_*]*T*. Finally in the DWT approach “db4” was used as the analyzing wavelet. Figure 10 reports the Precision and Recall scores (using the same multiclass SVM classifier) obtained when one of the above methods was used to pre-process the large array of acquired data: the better capability of the classifier to recognize the pure compound and the concentrations of binary mixtures when the proposed pre-processing strategy was used is evident.

**Figure 11.**
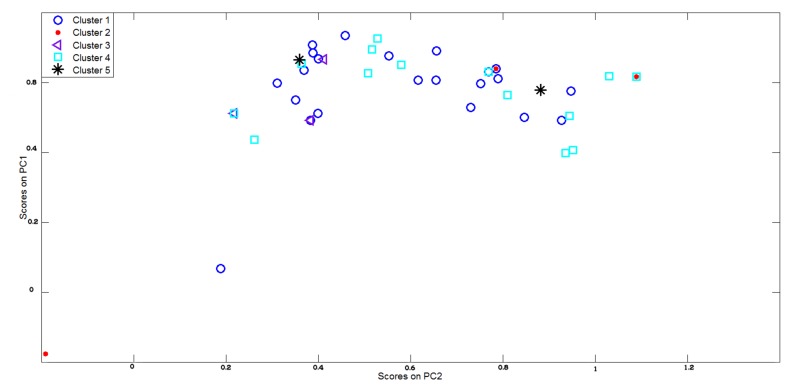
Data clusterization experienced by using k-means strategy on dataset 1.

To further highlight the difficulties behind the classification problem under consideration a final evidence has been given by using the k-means algorithm [35] to clusterize sensory outputs. Figure 11 reports the principal data directions as in Figure 6 but in this case the data points are differently marked since each colors and shape is associated to the cluster number supplied by the k-means algorithm. As shown in Figure 11, even if the number of clusters (*i.e.*, 5) is put as a priori knowledge, the clusterization results was very poor and it is impossible to recognize the different classes of substances in input to the sensor array.

**Figure 12.**
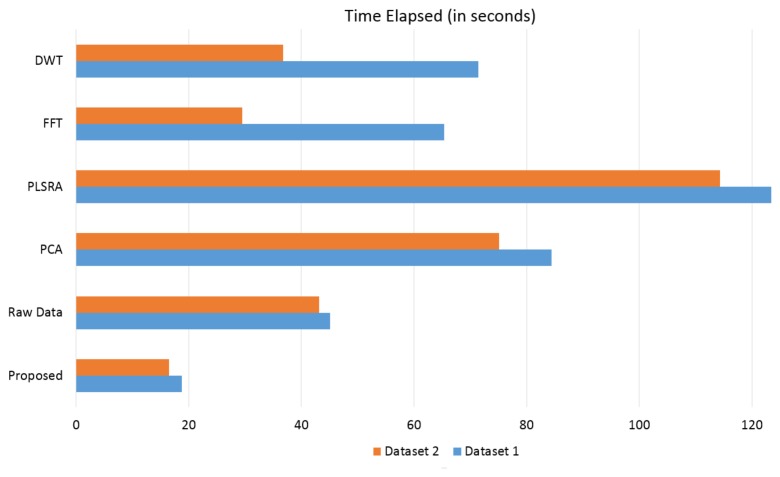
Elapsed time (in seconds) for the computation of the comparing solutions (pre-procesing and k-fold SVM classification).

Another aspect that should be considered when a large amount of data has to be handled, is the time required to complete the whole evaluation procedure. All techniques have been implemented using Matlab R2013b and they were run on a system equipped with an i7-3630QM CPU (2.40 Ghz) and 16Gb of RAM: the elapsed time (in seconds) for the computation of the comparing solutions (preprocessing and k-fold SVM classification) is graphically reported Figure 12. This demonstrated that the proposed solution is also more efficient in terms of computational load: it is thus more suited to be used in case of extensive tests of electronic device when, for example, different setups have to be experimentally evaluated.

## 7. Conclusions

An appropriate signal processing pipeline for a complex artificial olfactory system (Electronic Nose) has been introduced in this paper. The device consists of a Large-Scale Chemical Sensor Array that supplies data to the software modules which perform advanced data processing in order to automatically recognize pure analytes and/or their binary mixtures. The paper concentrated then on the software components that allow the transduction of the sensor outputs to an automatic assessment of the substances given as input. The proposed multistep strategy firstly normalizes the heterogeneous sensory data and then reduces their inherent noise. Cleaned data are finally supplied as input to a data reduction procedure that extracts the most informative and discriminant directions in order to get an efficient representation in a lower dimensional space in which graphical and analytic classification can be effectively performed. This work introduces a novel way to deal with a very large amount of data dimensionality and introduces the concept of EigenOdour as a mathematical procedure allowing data reduction under the Principal Component Analysis framework. Data separation is also addressed by finding directions of largest separation of clusters under the linear discriminant analysis framework. A classification stage with opportune basis representation is then performed with multi-class support vector machines. Experimental results on two different datasets demonstrating the effectiveness of the proposed approach to reduce dimensionality and to get a data representation more and more suited for classification/segmentation tasks are shown. This has been demonstrated by qualitative and quantitative analysis and the experimental outcomes represent an encouraging achievement towards an olfactory device able to mimic the biological olfactory system. Future study will deal with sparse data representation, to aid manifold learning using unsupervised approaches directly from acquired data. Moreover, algorithms to compensate the effect of the variation of humidity and temperature could be investigated.

## Figures and Tables

**Table 1.** The polymers deposited on the sensor arrays: their sensitivities were differentiated by means of functional groups chains attached to the main ring, by using a range of dopant species or preparing copolymers.

**Class Number**	**Polymer Type**	**Class Number**	**Polymer Type**
1	PANI/CB	14	PANI/CSA/PVP
2	PEDOT	15	PANI/pTSA/PS
3	PANI Long Chain	16	PANI/pTSA
4	PPY doped	17	PANI/CSA/PEO
5	PDDT/FeCL_3_	18	PANI/pTSA/PEO
6	PANIPOL	19	PANI/CSA/PS
7	P3HT	20	PANI/PTSA/PVP
8	PPY/CB	21	PANI/CSA/PMMA
9	PDDT/FeCL_3_ modified	22	PANI/DBSA/PS
10	PANI/CSA	23	PANI/DBSA/PMMA
11	P3DT/FeCL_3_	24	PANI/DBSA/PEO
12	P3OT/FeCL_3_	25	Poly(3-methyl-4-hexylthiophene-2,5-diyl)
13	P3BT/FeCL_3_		

**Table 2.** Odour proportions and concentrations during the collection of the dataset 1.

**Num. of Meas.**	**Odour's Composition**	**Odour's Label**
8	100% Butanone (19,520 ppm)	B100
8	75% Butanone (14,656 ppm) - 25% Ethanol (3433 ppm)	B75E25
9	50% Butanone (9760 ppm)- 50% Ethanol (6867 ppm)	B50E50
8	25% Butanone (4880 ppm)- 75% Ethanol (10,300 ppm)	B25E75
8	100% Ethanol (13,734 ppm)	E100

**Table 3.** Odour proportions and concentrations during the collection of the dataset 2.

**Num. of Meas.**	**Odour's Composition**	**Odour's Label**
8	100% Ethanol (13734 ppm)	E100
3	20% Butanone (4880 ppm)- 80% Ethanol (13,734 ppm)	B20E80
3	33% Butanone (9760 ppm)- 67% Ethanol (13,734 ppm)	B33E67
3	43% Butanone (14,656 ppm)- 57% Ethanol (13,734 ppm)	B43E57
3	50% Butanone (19,520 ppm)- 50% Ethanol (13,734 ppm)	B50E50

**Table 4.** Odour classification using *k*– *fold* cross validation during the collection of the Dataset 1.

	**B100**	**B75E25**	**B50E50**	**B25E75**	**E100**
B100	**8**	0	0	0	0
B75E25	0	**8**	2	0	0
B50E50	0	0	**7**	3	0
B25E75	0	0	0	**5**	0
E100	0	0	0	0	**8**

**Table 5.** Odor classification using *k* – *fold* cross validation during the collection of the dataset 2.

	**B10E40**	**B20E40**	**B30E40**	**B40E40**	**E40**
B10E40	**3**	0	0	0	0
B20E40	0	**3**	0	0	0
B30E40	0	0	**3**	0	0
B40E40	0	0	0	**3**	0
E40	0	0	0	0	**8**
